# Synthesis of Nanohydroxyapatite from Cuttlefish Bone (*Sepia *sp.) Using Milling Method

**DOI:** 10.1155/2019/1831208

**Published:** 2019-05-02

**Authors:** Aminatun, Adri Supardi, Zulifah Izzatin Nisa, Dyah Hikmawati, Siswanto

**Affiliations:** Department of Physics, Faculty of Science and Technology, Universitas Airlangga, Surabaya 60115, Indonesia

## Abstract

The synthesis of nanohydroxyapatite from cuttlefish bone (*Sepia *sp.) has been done by using High Energy Milling (HEM) and its characterization in vitro as bone repair. This study aimed to determine the effect of the milling process on microscopic properties and mechanical properties of nano-HA through XRD, TEM, and compressive strength tests. The hydroxyapatite (HA) used in this study consisted of 1M CaCO_3_ from aragonite which was extracted from cuttlefish lamella bone (*Sepia *sp.) and 0.6 M NH_4_H_2_PO_4_, which was hydrothermally processed at 200°C for 12 h and then sintered at 900°C for 1h. Parameter milling includes the variation of milling time, i.e., 3 h, 6 h, and 9 h at rotational speed of 350 rpm. An increase in milling time causes a decrease in HA particle size. This is shown from the results of TEM at the milling time of 9 h with the smallest size up to 65 nm. The result of cell viability test showed that all samples are not toxic with cell viability value of >80%. The milling time of 9 h was an optimum condition with a compressive strength of 4.35952 MPa that can be applied to cancellous bone.

## 1. Introduction

Hydroxyapatite (HA) is the largest component (70%) of the total phase of the bone mineral. HA contains calcium and phosphorus ratio similar to natural bone which is 1.67. HA with the chemical formula [Ca_10_(PO_4_)_6_(OH)_2_] is one of the most effective bone repair material used in the orthopaedic field to repair parts of bone that are damaged by accident or disease [1]. In addition to corrosion resistance, HA is also bioactive which also means that it can form a linkage between interfaces of the material with body tissue and between two bones. HA is a biocompatible material that does not react with other body parts and can merge with bone. Its function is very diverse; i.e., it can serve as a bone filler, as a porous scaffold, or as a material coating prosthesis [2–8].

Synthesis of HA made from cuttlefish bone has been done via hydrothermal method and followed by a sintering process [3, 9–12]. The dorsal and lamellae parts of* Sepia *sp. were separated and heated at 350°C for 3h. The result of XRD showed that aragonite CaCO_3_ content originating from dorsal part became calcite CaCO_3_. Thus, the synthesis of hydroxyapatite used the lamellae part of* Sepia* sp. The aragonite CaCO_3_ changed easily and transformed into hydroxyapatite after the addition of NH_4_H_2_PO_4_ through hydrothermal method at 200°C for 24 hours [12]. The best result is shown with the crystal structure, the grain morphology, the toxicity, and the compressive strength occurring in the hydrothermal process at 200°C for 12h, followed by the sintering process at 900°C for 1h. However, the size of HA particles generated was still in microorder [3].

Nanometer-sized particles can accelerate osteoconduction and osteointegration processes in bone tissues. The proportion of atomic surface of nanosized particles will be better [13]. When it is compacted on nanosized materials, the structure of material will be more compact than the microsized material. Synthesis of nano-HA via the milling method was done with a rotational speed of 530 rpm and a time variation of 2h to 40h [14]. The results obtained showed that nanosized HA with good characteristics and suitability with the bone apatite have been produced for more than 12h. The nanosize of HA increases with an increase of the milling time.

Milling technique using High Energy Milling (HEM) is a destructive technique that makes the surface area of a sample becomes wider, accompanied by the collision energy between the milling balls and the rotating vial wall so that the particle size of the resulting sample is smaller. The milling parameters that influence the milling process include the type of milling tool, the ratio between ball and sample, the size and the type of ball material, the milling time, the milling speed, the milling temperature, the sample type, the initial material size, and the material type of vial. The advantage of using HEM is that it produces large strains and nanocrystal structures [15].

Based on the description stated above, this study was conducted to make the nano-HA from the cuttlefish bone with variations of milling time to produce a suitable nano-HA powder for the bone repair application.

## 2. Materials and Methods

This research was conducted in two stages: (1) synthesis of HA via hydrothermal method with Ca precursor extracted from cuttlefish bone and (2) milling process by using HEM to produce a nanosized HA powder.

### 2.1. Synthesis of HA via Hydrothermal Method followed by Sintering Process

The process of synthesis began by extracting CaCO_3_ from the cuttlefish bone by taking the lamellae part of the cuttlefish bone and making it a powder using mortar. Then the powder was heated in a furnace at 350°C for 3 h. The synthesis of HA was obtained by mixing 10g CaCO_3_ into 100 ml of water to get 1M CaCO_3_ solution, while 0.4M NH_4_H_2_PO_4_ solution was made by dissolving 6.9g NH_4_H_2_PO_4_ into 100 ml of water. The reaction equation between those compounds is

(1) 10CaCO3+6NH4H2PO4+2H2O→Ca10PO46OH2+3NH42CO3+7H2CO3

1M CaCO_3_ extracted from the lamellae part of the cuttlefish bone was mixed with 0.6M NH_4_H_2_PO_4_ (Merck, 99.9+) using a magnetic stirrer for 30 min. After that, the mixture was transferred to a SS 316L reactor and heated in an electric oven at 200°C for 12 h. Then the sample was cooled at room temperature. Furthermore, the sample was washed with water and rinsed repeatedly to remove other acidic products until neutral pH was reached. The final rinse was done using methanol p.a. to limit the agglomeration of HA particles during the drying process. Then the rinsed sample was filtered using filter paper and dried in an electric oven at 50°C for 1 h. The HA powder formed was then sintered at 900°C for 1 h by using a furnace [3].

### 2.2. Synthesis of Nano-HA via Milling Method

HA powder resulting from the previous step was then given a milling treatment with ratio mass of HA powder: ball milling mass of 1: 20 and milling times of 3h, 6h, and 9h, which were, respectively, named as Q3, Q6, and Q9. After that, HA powder and ball milling made of alumina were weighed according to the ratio then put into the vial milling. The milling time was applied repeatedly until the accumulated time was obtained as specified.

### 2.3. Sample Characterization

The XRD pattern was recorded at the angular range of 2*θ* = 5°-60°. In this experiment, Xpert-Pro Analytical diffractometer (30 mA, 40 kV) with the K_*α*1_, radiation of copper (*λ* = 1.5406 Å), was used. From the observation of XRD samples, it resulted in spectra at peak intensities with a certain angle of 2*θ*. Then this result was matched with ICDD data to identify the resulting diffraction peak. The degree of crystallinity of nanostructured HA was calculated by

(2) $$\begin{array}{c} Xc=1-\left(\;V_{112/300}-I_{300}\;\right) \end{array}$$

In (2), Xc is the degree of crystallinity; V_112/300_ is the depth of the valley between the characteristic peaks corresponding to the planes of (112) and (300); and I_300_ is the intensity of (300) planes. In another way, the percent of crystallinity was calculated by the areas under the peaks of XRD (A_crytalline_ and A_amorphous_ are the areas under the XRD pattern of crystalline and amorphous parts). This calculation was done by the Origin 70 software which can be calculated with

(3) Percent  of  Crystallinity=AcrytallineAcrytalline+Aamorphous×100%

The crystallite size and the lattice strain of HA powder after mechanical alloying can be estimated by Williamson-Hall method [16].

(4) $$\begin{array}{c} b\cos\theta =\frac{0\text{,}9\lambda }{d}+2\eta \sin\theta \end{array}$$

where d is the grain size, *λ* is the wave length of used X-ray, b is the full width at half height FWHM, *θ* is the Bragg diffraction angle, and *η* is the crystalline lattice strain.

The lattice parameter of hexagonal system of HA can be calculated by Powder Cell of Window (PCW) program with

(5) $$\begin{array}{c} \frac{1}{d^{2}}=\frac{4}{3}\left(\;\frac{h^{2}+hk+k^{2}}{a^{2}}\;\right)+\frac{l^{2}}{c^{2}} \end{array}$$

where d_hkl_ is the distance between hkl indexed fields and hkl is the Miller index. As for a, b, c, they are lattice parameters, and n is a shared factor that divides the indexed field into the smallest integer. The lattice parameters are closely related to the volume of crystal. The volume of the hexagonal crystal was calculated by

(6) $$\begin{array}{c} V=a^{2}c\sin60^{{}^{\circ}} \end{array}$$

The characterization of particle size and grain morphology was performed by using TEM with a magnification of 10.000. First, the originally powder-formed samples were prepared by dissolving them into ethanol; then the ultrasonication process was done to the solution to make it homogeneous. Furthermore, a small golden chip with a diameter of 1-2 mm was put into the sample solution; then it was dried. Following that, the chip was put into a cylinder-shaped holder. The cylinder-shaped holder was then inserted into the TEM tool after the TEM tool had been vacuumed. Next, the most clear and best figure displayed on the screen was taken. The result of selected figure was processed in a computer program which was directly connected to the TEM tool.

The compressive strength was characterized by using Shimadzu Autograph AG-10TE. The sample was printed in advance until it became pellet-shaped by compacting it by using 4 tons weight. The sample weighed 2g with the diameter of 15 mm. Then the sample was placed on the press machine; then the engine was turned on and the speed and the force to be measured were also set. Next, the load cell was lowered slowly; then it was stopped. The resulting force and strain were then written. This step was done with very small changes until the sample was broken and the maximum force that the sample can retain was shown on the press machine's display. The obtained quantities from this test were then calculated by using (7) to find out the compressive strength of the sample.

(7) $$\begin{array}{c} \sigma =\frac{F}{A} \end{array}$$

where *σ*= stress (N/m^2^); F= force (N); and A= cross-sectional area of sample (m^2^).

The cell viability testing was conducted by using MTT* Assay* consisting of tetrazolium salt [3-(4,5-dimetiltiazol-2-yl)-2,5-difeniltetrazolium bromide]. The systematic principle of MTT* Assay *method is based on the living cell ability which can be seen from the mitochondrial activities of cell culture. The cell used is fibroblast cell from Cell Line BHK-21. The basic method of MTT Assay is the changes occurring from tetrazolium salt [3-(4,5-dimetiltiazol-2-yl)-2,5-difeniltetrazolium bromide] to formazan in an active mitochondria which was read by Elisa reader with wavelength of 570 nm. The living cell will change MTT which was then cracked through the reduction of reductase enzyme in a chain of mitochondrial respiratory system to formazan which was dissolved in purple. The bigger the absorbance, the more the living cells which can be calculated by using the following:

(8) $$\begin{array}{c} \%\;Cell\;\;Viability=\frac{ODT-ODM}{ODC-ODM}\times 100\% \end{array}$$

where ODT is OD treated cells, ODM is OD media control, and ODC is OD cells control.

## 3. Results and Discussion

### 3.1. The Results of XRD Test of HA Powder after and before the Milling Process

**Figure 1:**
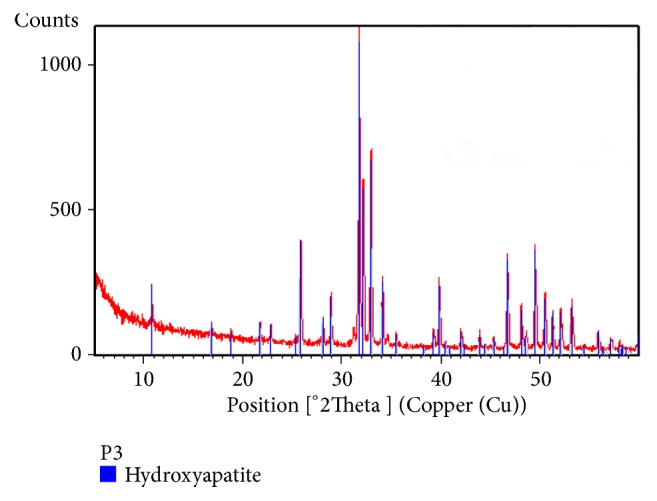
XRD spectrum of cuttlefish bone-derived HA (*Sepia *sp.).

The HA, which was synthesized through a hydrothermal process at 200°C for 12h and then sintered at 900°C for 1h (control sample of HA), was characterized with XRD test at short angle of 2*θ* = 5°-60°, which is shown in Figure 1, with the highest intensity peak at 2*θ* = 31.8807° of 1684.54. The identification results showed 100% compatibility with ICDD 01-074-0565 reference data for HA (Ca_10_(PO_4_)_6_(OH)_2_).

**Figure 2:**
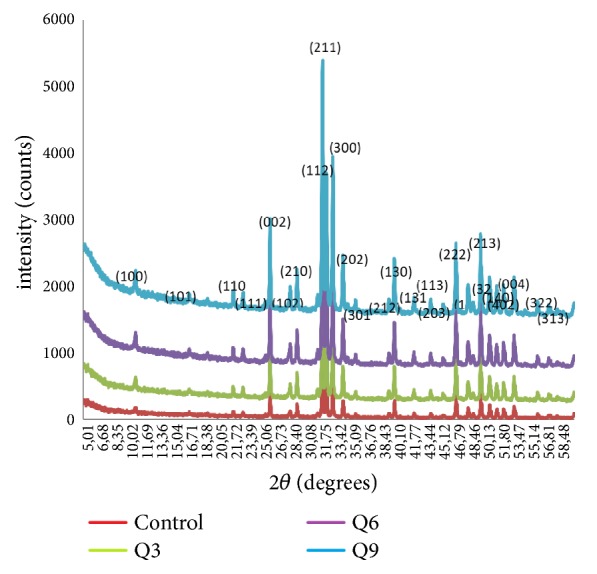
XRD spectrum of HA after milling process.

Furthermore, the HA from hydrothermal and sintering process was milled for 3 h, 6 h, and 9 h with ratio of powder mass: milling ball mass of 1:20. Then resintering process was done at 900°C for 1 h. Next, the results were characterized by XRD test and followed by phase identification. The result can be seen in Figure 2. It shows that all figure peaks can be identified as HA with a 100% level of conformity to ICDD 01-074-0565. The maximum diffraction peak of HA after milling is presented in Table 1.

The degree of crystallinity (initially around 84%) decreased along with the increase of milling times of 3, 6, and 9 h; with each of milling time, the degree of crystallinity became 82%, 77%, and 81%) (see (2)). Calculation of the area under the XRD peak showed that the percent of crystallinity was around 75% (still needs to be checked with the origin). The milling process decreased the degree of crystallinity. This is because the milling process creates a grid strain that can be proven on the graph made based on (4).

**Figure 3:**
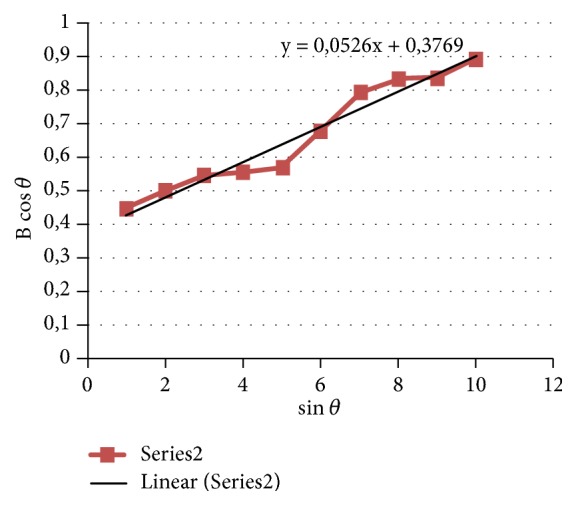
Williamson-Hall plot of HA powder after 3 h of milling.

According to (4), the plot of b cos⁡*θ* versus 2sin⁡*θ* gives a straight line with a slope equal to *η* and the intercept along y-axis as 0.9*λ*/d.  Figure 3 shows the method of Williamson-Hall equation for estimating crystallite size and lattice strain of HA powder after 3h of milling. So, the crystallite size of HA was approximately around 36.79 nm and the lattice strain was 0.0526%. The presence of crystal lattice strain of HA due to milling was also seen in the shift of the maximum diffraction peak angle of 2*θ* from the initial position before the milling process of 31.8807° became 31.7650°.

In this research, refinement via PCW program was conducted. The information obtained is the lattice parameter, as presented in Table 2.

Table 2 shows the change of lattice parameter values of a and c. The four samples have lattice parameter values that are close to the reference lattice parameter of HA; i.e., a = b = 9.424 Å and c= 6.879Å. The value of the lattice parameter is related to the volume of crystal that can be calculated by (6).

### 3.2. Transmission Electron Microscope (TEM)

**Figure 4:**
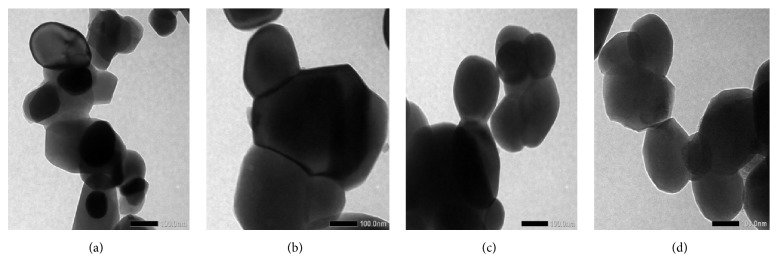
TEM test results of sample HA: (a) control, (b) Q3, (c) Q6, and (d) Q9.

The analysis of morphology and particle size was obtained from TEM test results with a magnification of 10,000x to 40.000x. The results of this characterization can be seen in Figure 4.

The TEM results in Figure 4 show the nonhomogeneous morphology of samples with irregular spherical particles and the nonhomogeneous particle distribution. The particles overlapped each other to form larger clusters. Particle size and cluster size data from TEM test results are presented in Table 3.

Table 3 show the samples after the sintering process, which indicate that the longer the milling time the smaller the particle size produced. The particle sizes of HA after the milling process showed a significant decrease that made the sizes included in the size range of a nanomaterial.

### 3.3. Compressive Strength

**Figure 5:**
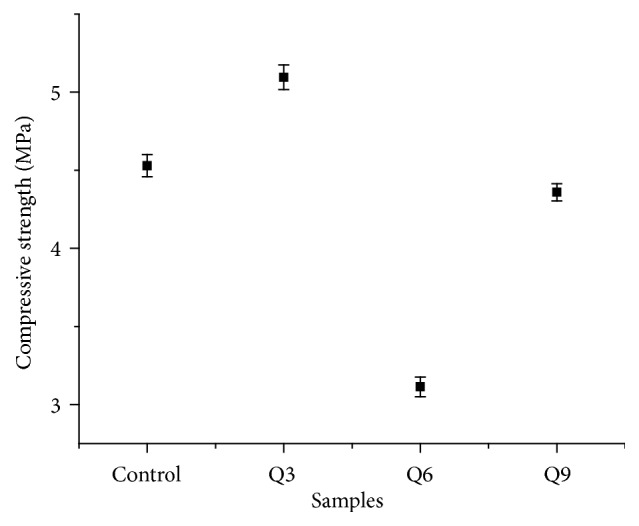
Compressive strength.

The compressive strength was obtained from (7), which was presented in Figure 5.

In Figure 5, the compressive strength was influenced by the particle size and the size of the cluster formed. The compressive strength values are inversely proportional to particle size and cluster. The smaller the particle size and the cluster on the material, the greater the compressive strength. The particle size affects the compaction process of samples so that it affects the compressive strength as well. When the particle size of nanoordered HA is compacted, the particles become more compact and neatly arranged that it increases the compressive strength. The compressive strength of all samples ranged from 3.11394 to 5.09554 MPa, which is suitable for application on cancellous bone.

### 3.4. Cell Viability

**Figure 6:**
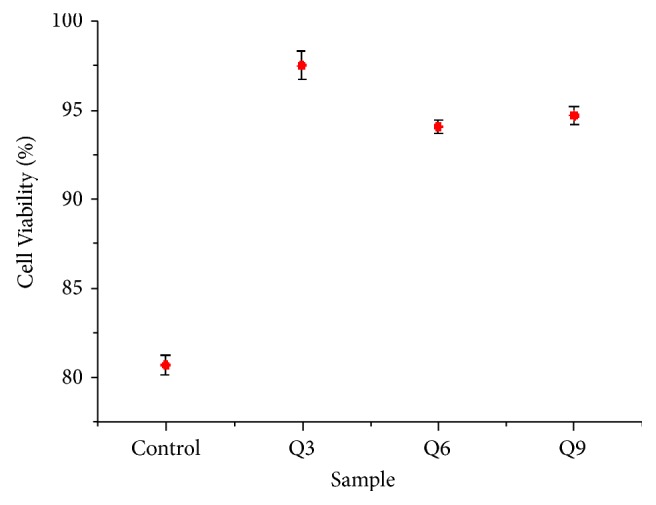
Percentage of cell viability.

Cell viability test aims to know the nature of cytotoxicity in the nano-HA sample. A material is called nontoxic if the percentage of its cell viability is more than 50% [17, 18]. From the cell viability test using MTT Assay, the results obtained showed that the cell viability percentage for nano-HA samples of control, Q3, Q6, and Q9 groups are 80.72%, 97.46%, 94.05%, and 94.66%, respectively (Figure 6). These results show that the HA sample (control) is not toxic and so are the nano-HA samples from Q3, Q6, and Q9. HA is osteoconductive, which can trigger the growth of osteoblast bone cells. Bone cell growth due to the presence of HA starts from the dissolution of HA by releasing calcium and phosphate in body fluids, resulting in carbonate precipitation because HA is bioactive.

Based on the Kolmogorov-Smirnov normality test with *α* = 0.01, all samples, namely, control, Q3, Q6, and Q9, are normally distributed with the Sig. > 0.01. Furthermore, a difference test was carried out using paired sample t-tests which showed that the treatment of Q3, Q4, and Q6 is significantly different from control. Each Sig. (2-tailed) value is 0.000 <0.01, which means that the non-nanosize, temporarily between Q3 and Q6, Q3 with Q9, and Q6 with Q9, is not significantly different because the Sig. (2-tailed) values are 0.521, 0.606, and 0.874, respectively. This also indicates that different nanosizes do not affect the level of cell viability.

## 4. Conclusions

It can be concluded that the milling process using HEM at 350 rpm with milling time variations of 3h, 6h, and 9 h affects the crystallinity, the particle size, and the compressive strength of HA. Longer milling times lessen the crystallinity and the particle size of HA. The particle size obtained ranged between 65.26 nm and 216.60 nm while the cluster size ranged between 254.25 nm and 847.68 nm. The optimal milling time variation is found at Q9 sample with the milling time of 9h that produced the particle size of 65.26 nm to 200.20 nm and the compressive strength of 4.35952 MPa. The cell viability test results showed that all samples were not toxic with a cell viability value > 80%. The result of Q9 sample is found to be the most suitable as a cancellous bone implant material.

## Figures and Tables

**Table 1:** Maximum diffraction peak of HA.

Sample	Maximum Diffraction Peak	
	2*θ* (°2theta)	Intensity
Control	31.8807	1684.54

Q3	31.7930	1169.90

Q6	31.8153	723.41

Q9	31.7845	784.57

**Table 2:** Lattice parameter of HA.

Parameter	ICSD	Samples			
		HA (control)	Q3	Q6	Q9
Rp	-	19.84	15.46	15.01	14.45

Rwp	-	25.84	21.26	20.03	19.93

Rexp	-	3.11	3.02	2.01	2.29

a=b	9.424	9.410998	9.422140	9.425058	9.420666

C	6.879	6.864786	6.879305	6.879669	6.880002

Volume of crystal	529.07	526.52	528.89	529.24	528.77

**Table 3:** Particle and cluster sizes taken from TEM test.

Sample Name	Particle Size	Cluster Size
	(nm)	(nm)
Control	91.48-150.81	209.79 - 394.28

Q3	216.60-378.03	334.46 - 847.68

Q6	127.91-184.07	290.09 - 323.69

Q9	65.26-200.20	254.25 - 389.15

## Data Availability

The data used to support the findings of this study are included within the article.
